# The Osteoblastogenesis Potential of Adipose Mesenchymal Stem Cells in Myeloma Patients Who Had Received Intensive Therapy

**DOI:** 10.1371/journal.pone.0094395

**Published:** 2014-04-10

**Authors:** Hsiu-Hsia Lin, Shiaw-Min Hwang, Shang-Ju Wu, Lee-Feng Hsu, Yi-Hua Liao, Yi-Shuan Sheen, Wen-Hui Chuang, Shang-Yi Huang

**Affiliations:** 1 Department of Internal Medicine, National Taiwan University Hospital, Taipei, Taiwan; 2 Bioresource Collection and Research Center, Food Industry Research and Development Institute, Hsinchu, Taiwan; 3 Department of Dermatology, National Taiwan University Hospital, Taipei, Taiwan; Josep Carreras Leukaemia Research Institute, University of Barcelona, Spain

## Abstract

Multiple myeloma (MM) is characterized by advanced osteolytic lesions resulting from the activation of osteoclasts (OCs) and inhibition of osteoblasts (OBs). OBs are derived from mesenchymal stem cells (MSCs) from the bone marrow (BM), however the pool and function of BMMSCs in MM patients (MM-BMMSCs) are reduced by myeloma cells (MCs) and cytokines secreted from MCs and related anti-MM treatment. Such reduction in MM-BMMSCs currently cannot be restored by any means. Recently, genetic aberrations of MM-BMMSCs have been noted, which further impaired their differentiation toward OBs. We hypothesize that the MSCs derived from adipose tissue (ADMSCs) can be used as alternative MSC sources to enhance the pool and function of OBs. Therefore, the purpose of this study was to compare the osteogenesis ability of paired ADMSCs and BMMSCs in MM patients who had completed intensive therapy. Fifteen MM patients who had received bortezomib-based induction and autologous transplantation were enrolled. At the third month after the transplant, the paired ADMSCs and BMMSCs were obtained and cultured. Compared with the BMMSCs, the ADMSCs exhibited a significantly higher expansion capacity (100% vs 13%, respectively; *P* = .001) and shorter doubling time (28 hours vs 115 hours, respectively; *P* = .019). After inducing osteogenic differentiation, although the ALP activity did not differ between the ADMSCs and BMMSCs (0.78 U/µg vs 0.74±0.14 U/µg, respectively; *P* = .834), the ADMSCs still exhibited higher calcium mineralization, which was determined using Alizarin red S (1029 nmole vs 341 nmole, respectively; *P* = .001) and von Kossa staining (2.6 E+05 µm^2^ vs 5 E+04 µm^2^, respectively; *P* = .042), than the BMMSCs did. Our results suggested that ADMSCs are a feasible MSC source for enhancing the pool and function of OBs in MM patients who have received intensive therapy.

## Introduction

Multiple myeloma (MM), a malignant B-cell disorder, is characterized by the accumulation of neoplastic plasma cells within bone marrow (BM) and punched-out (lytic) bone lesions [1]. Advanced lytic bone lesions and related pathologic fractures affect nearly 50% of MM patients throughout their disease courses, and are critical clinical concerns that result in 20% higher mortality compared with MM patients without fractures [2]. MM-related bone destruction (MMBD) is caused by increased activity of osteoclasts (OCs) which, however, causes bone loss with concomitant loss of bone repair and growth from the suppression of osteoblasts (OBs) [2]. Myeloma cells (MCs) secrete several cytokines, including the receptor activator of NF-κB ligand (RANKL), interleukin-3 (IL-3), IL-6, and activin A to activate osteoclasts (OCs), and secrete dickkopf-1 (DKK1) and soluble frizzled related proteins (SFRPs) to inhibit the differentiation and maturation of osteoblasts (OBs) [2]–[7].

Current therapeutic options for MMBD include bisphosphonates, radiotherapy, and surgery, which may reduce bone pain, the development of new osteolytic lesions, and prevent skeletal-related events (SREs), including pathologic fractures [2]. But only the bisphosphonates are able to inhibit OCs substantially, the effect of radiotherapy and surgery, however, is mainly based on an antitumor and symptomatic effect without an evident role in the modification of the function of OCs. These treatments for MMBD, no matter bisphosphonates or radiation and surgery, do not enhance either the pool or function of OBs; in other words, they do not improve osteoblastogenesis in MM patients [2], [4]. It is the reason why in most clinical scenarios, MMBD seldom heal despite adequate treatment, even when the disease is in remission [2], [8]. In physiological conditions, OBs are derived from mesenchymal stem cells (MSCs) that reside in the BM [9]. However, human MSCs also reside in various tissues other than the BM and are able to differentiate into multiple tissue lineages, including cartilage and bone [10]–[13]. Numerous studies on using MSC-related therapy in the regeneration of musculoskeletal and neural tissues and the recovery of endothelial function have been conducted [14], [15]. In MMBD, MSCs derived from the BM (BMMSCs) have been used to heal bone lesions [16]. However, recent studies have shown that BMMSCs from MM patients (MM-BMMSCs) have several aberrant gene expressions and chromosomal abnormalities compared with those from healthy donors [17]–[19].The origin of these genetic aberrations in MM-BMMSCs is unclear, but the genetic aberrations seemed to persist in the MM-BMMSCs even though the clinical remission of MM was achieved through adequate treatment [19]. Certain aberrant regulatory genes, such as the bone morphogenetic protein (BMP) family have been associated with osteogenic differentiation [17], [19], [20]. However, according to our thorough review of relevant literature, efforts to repair lytic bone lesions in MM patients by using MM-BMMSCs have not yet succeeded [2], [6]. Therefore, we hypothesize that an alternative MSC other than BMMSCs in MM patients can be used as an effective cellular source for repairing osteolytic bone lesions in the future. Adipose-tissue-derived MSCs (ADMSCs) are candidates for repairing osteolytic bone lesions because they reside outside the BM, where the microenvironment might not have been altered by the MCs, and exhibit osteogenic potential [21], [22]. Compared with BMMSCs, ADMSCs have similar self-renewal ability [21] and can differentiate into other mesodermal lineages [21], [23]. Furthermore, a recent study showed that osteogenesis ability of ADMSCs is less affected by repeated cell passages and donor age than that from BMMSCs [24], indicating that ADMSCs are an ideal alternative MSC source for osteoblastogenesis in MM patients.

The purposes of this study were to compare the osteogenesis potential of paired BMMSCs and ADMSCs obtained from MM patients who completed a bortezomib-based induction regimen followed by high-dose chemotherapy with autologous stem cell transplantation (HDT/AuSCT), a standard treatment for MM patients [1], and to determine whether ADMSCs are a suitable MSC source that can differentiate into adequate and functional OBs in MM patients.

## Materials and Methods

### MM Patients

From July 2012 to August 2013, 15 MM patients who had received a bortezomib-based induction regimen followed by HDT/AuSCT (200 mg/m^2^ of melphalan) were enrolled. The dosage and schedule of the treatment were similar to those reported previously [25]. In brief, bortezomib (1.3 mg/m^2^) was administered on Days 1, 4, 8, and 11 in a 21-day cycle, thalidomide (200 mg) was administered daily, and dexamethasone (80 mg) was administered weekly. Seven patients received additional cyclophosphamide (100 mg daily) for the first 4 days in each cycle. One of patient had received doxorubicin (9 mg/m^2^ i.v.f. for consecutive 4 days) instead of thalidomide due to personal intolerance.

### Paired bone marrow samples and subcutaneous adipose tissues and controls

Paired BM samples and subcutaneous adipose tissues were obtained from eachMM patient at the regular BM examination approximately 3 months after Day 0 of the HDT/AuSCT. Subcutaneous adipose tissues (sizes 0.3–0.5 mm^3^) under the BM puncture site were obtained by performing surgical exploration. During the same period of time, bone marrow samples obtained from 5 non-MM patients, including stage I lung cancer (1), perivascular lympocytic dermatitis (1), and stage I/II lymphoma (3) with a median age of 69 years (range: 39–84 y), were used as a non-MM control; while subcutaneous adipose tissues biopsied from another 4 healthy donors with a median age of 38 years (range 27–73 y) were also obtained. This study was approved by National Taiwan University Hospital Research Ethics Committee (NTUHREC: 201106013RC), and written informed consent was obtained from each study subject in accordance with the Declaration of Helsinki.

### Isolation of bone marrow mesenchymal stem cells and adipose tissue-derived mesenchymal stem cells and the cell culture

BM mononuclear cells (BMMNCs) were obtained by conducting equal-volume Ficoll centrifugation (2000 rpm, 40 minutes) (GE Healthcare, UK). The BMMNCs were then seeded in a flask at a cell density of 2×10^6^/cm^2^. The basic growth medium consisted of α-MEM (GIBCO, CA, USA)+20% FBS (Corning, NY, USA)+4 ng/mL of bFGF (Millipore, MA, USA)+1xPS (GIBCO, CA, USA). The paired subcutaneous adipose tissues were dissected into small pieces and digested with collagenase IV (SERVA Electrophoresis GmbH, Heidelberg, Germany) and hyaluronidase (Sigma-Aldrich, MO, USA), and were cultured in the basic growth medium (α-MEM+20% FBS+4 ng/mL bFGF+1xPS) at 37°C for 50 minutes with intermittent shaking. The resulting suspensions were filtered using a 70-µm strainer (BD Bioscience, NJ, USA) to remove debris and then centrifuged at 2000 rpm for 10 minutes. The supernatants were discarded, and the cell pellets were resuspended in the basic growth medium and cultured at density of 1.3×10^5^/cm^2^ in a tissue flask (37°C, 5% CO_2_). The basic growth medium was changed every 3 days and the cells were maintained at subconfluent levels (Passage 0, P0). The attached cells (BMMSCs and ADMSCs) were then harvested using trypsin-EDTA (GIBCO, CA, USA), and were subcultured at a density of 2–3×10^3^ cells/cm^2^ under the same conditions used in the primary culture (Passage 1, P1).

### Flow cytometry

Flow cytometry was performed by employing a FACSVerse flow cytometer (BD Biosciences, NJ, USA) and using a panel of monoclonal antibodies to identify MSCs, namely CD105-PE, CD73-PE, CD44-PE, and CD90-PE (BD Biosciences, NJ, USA) and hematopoietic cells, namely CD14-FITC, CD19-FITC (BD Biosciences, NJ, USA), CD45-FITC/CD34-PE (Thermo Fisher Scientific, MA, USA) [26], [27], CD138-FITC and HLA-DR (BD Biosciences, NJ, USA) [28], as well as isotype-matched controls (BD Biosciences, NJ, USA).

### Proliferation assay

The proliferation potential of the cultured MSCs from the second passage (P2) to the fourth passage (P4) was evaluated according to the cumulative population doubling level (CPDL) as described previously [29], which was calculated as ln(Nf/Ni)/ln2, where Ni, Nf, and ln are initial seeded cell numbers, final cell numbers on the day of the subculture, and the natural log, respectively. The doubling time (hours) was then obtained by dividing 24 hours by the calculated CPDL.

### Expansion capacity of the cultured mesenchymal stem cells

As described previously [29], when the doubling time of the cultured MSCs was greater than 100 hours for a specific passage, the culture was considered to fail. The probability of expansion capacity of the cultured MSCs was estimated by the Kaplan-Meier survival curve, considering that the failure of proceeding culture at the specific passage was an event.

### Senescence associated beta-galactosidase staining

Ten thousand MSCs cells were plated in a 24 well plate for 24 h before the staining procedure. The detection of cellular senescence was performed by using the Senescence Detection Kit (Chemicon, USA & Canada) according to the manufacturer's instructions. Briefly, the cells washed with PBS at least 3 times and then fixed by fixation buffer at room temperature for 15 min. Rinsed and washed cells by PBS at least 3 times then stained by beta-galactosidase staining solution and incubated at 37°C overnight in a dry incubator (no CO_2_). Under a light microscopic examination, the blue granules developed within the cytoplasms were considered positive for the beta-galactosidase staining, suggesting senescence of the observed cells.

### Osteogenic differentiation

The osteogenic differentiation of the cultured MSCs from the second passage (P2) to the fourth passage (P4) was analyzed. The cells were seeded at 5×10^4^ cells/well in a 6-well plate (5×10^3^/cm^2^) and maintained in the basic growth medium until the cells reached 80% confluence. To induce osteogenic differentiation, the culture medium was changed to an osteogenic medium [α-MEM (GIBCO, CA, USA)+10% FBS (Corning, NY, USA)+1xPS (GIBCO, CA, USA)+10 nM dexamethasone (Dex, Sigma-Aldrich, MO, USA), 50 µg/mL of ascorbic acid (Sigma-Aldrich, MO, USA) and 10 mM β-glycerophosphate (Calbiochem, CA, USA)].

### Alkaline phosphatase activity

Alkaline phosphatase (ALP) activity of MSCs from passage 2 to 4 each was measured on the Day 4 by using the SensoLyte *p*NPP alkaline phosphatase assay kit (ANASPEC, Fremont, CA, USA). Briefly, the cells were lysed using an assay buffer with Triton X-100 and then centrifuged at 2500×*g* for 10 min at 4°C. The standard curve of ALP was generated using a serial dose of ALP (0–10 ng/well) and incubated with an ALP substrate solution (pNPP) at room temperature for 1 hour in each experiment. The detection range of ALP was 0 to 10 U. The cell lysates were incubated with an ALP substrate solution (pNPP) at room temperature for 1 hour, the reaction was stopped using a stop solution, and the absorbance was read at 405 nm (Perkin Elmer, CA, USA). The ALP activity (U) of cell lysates was calculated according to the ALP standard curve. The obtained ALP activity was then normalized according to the total protein (µg) in the loaded cell lysates and presented as U/µg. The total protein of cell lysates was determined using the Pierce™ BCA protein assay (Thermo Fisher Scientific, IL, USA).

### Alizarin red S staining

The cell matrix layer was washed with PBS, fixed with 70% cold ethanol, and stained with a 2% Alizarin red S (ARS) solution with a pH of 4.3 (Sigma-Aldrich, MO, USA) for 15 minutes. The amount of matrix mineralization was determined by dissolving the cell-bound ARS in 10% (w/v) cetylpyridinium chloride (CPC, Sigma-Aldrich, MO, USA) and 10 mM sodium phosphate, pH 7.0, and the standard curve of ARS was generated using a serial dose of ARS (0–100 nmole) in a 10% CPC solution. The absorbance was read at 562 nm (Perkin Elmer, CA, USA). The quantitative measurement of ARS absorbance in MSCs was calculated according to the generated standard curve for ARS.

### Bone nodule formation and von Kossa staining quantification

Corning Osteo Assay Surface multiple-well plates (Corning, NY, USA) were used to directly assess the osteoblastic activity in vitro as described previously [30], [31]. In brief, the MSCs were seeded at 2×10^4^/well (1×10^4^/cm^2^) in a Corning Osteo Surface plate (Corning, NY, USA) with the osteogenic medium. The osteogenic medium was refreshed every 3 days. The formed bone nodules were determined on Day 18 after osteogenic differentiation commenced and were measured using von Kossa staining. Briefly, cells were washed with PBS 3 times and fixed with 4% paraformaldehyde in PBS for 45 minutes at room temperature. After washing with deionized water, the cells were stained with 5% silver nitrate (Sigma-Aldrich, MO, USA) for another 45 minutes at room temperature under a bright light. To stop the silver nitrate reaction, the cells were washed with water and treated with a 5% solution of sodium thiosulfate (Sigma-Aldrich, MO, USA). Following another water wash and air drying, the nodules were visualized as dark staining patches under a light microscope. The area of von Kossa-positive nodules was determined using the public Image J software (developed by NIH, rsb.info.nih.gov/ij/) and the total areas (µm^2^) of the von Kossa-positive nodules were calculated.

### Statistics

Chi-square or Fisher's exact tests were used for between-group comparisons of the discrete variables. A Student paired *t* test or one-way ANOVA was used for between-group comparisons of the means. Kaplan-Meier survival curve was constructed to estimate the probability of expansion capacity, as a time to event defined by failure of proceeding further cultures of MSCs cultures, and the differences between groups were compared using the log-rank test. All directional *P* values were 2-tailed, and a value of .05 or less was considered significant for all tests. All analyses were performed using SPSS 18.0 software (Chicago, IL, USA).

## Results

### MM Patients

Seven men and 8 women with a median age of 57.5 years (range: 46–67 y) participated in this study. The clinical salient characteristics, treatment content, and response of these patients are summarized in Table 1. The overall response rate, terms of partial response or better, was 100% after the HDC/AuSCT, and the median induction duration was 8 months (range, 3–17 mo).

**Table 1:** The salient clinical characteristics at diagnosis and treatment response of the 15

Pt	Sex/age (yr)	Stage (DSS/ISS)	M-protein	PC in BM (%)	CA	Osteolytic lesion	Pathologic fracture	ZA	Induction regimen	Induction duration** (m)	Best Response* after HDT/AuSCT
1	F/51	II/II	IgD/kappa	80	no	yes	yes	yes	V+T+C+D	5	PR
2	M/51	IIIb/III	IgD/lambda	90	no	yes	yes	yes	V+T+D	10	CR
3	F/55	II/I	kappa	40	no	no	yes	yes	V+T+C+D	11	CR
4	M/38	I/I	IgA/lambda	5	no	no	yes	yes	V+T+D	8	CR
5	M/57	IIIb/III	IgG/kappa	92	no	yes	yes	yes	V+T+D	8	PR
6	F/60	II/I	IgG/kappa	60	no	no	no	yes	V+T+C+D	13	PR
7	F/58	II/I	IgG/kappa	30	no	yes	no	yes	V+T+C+D	9	CR
8	M/54	IIIa/II	IgG/lambda	30	no	no	no	yes	V+A+D	8	VGPR
9	F/53	II/II	IgG/lambda	50	no	no	no	no	V+T+D	8	VGPR
10	M/45	I/I	IgA/kappa	10	no	yes	yes	no	V+T+D	6	PR
11	F/47	IIIa/III	IgG/kappa	90	no	yes	no	no	V+T+D	3	VGPR
12	F/49	IIIa/II	IgG/kappa	90	yes	yes	yes	yes	V+T+D	5	CR
13	M/63	I/II	IgG/lambda	20	no	yes	no	yes	V+T+C+D	5	CR
14	F/59	IIIa/II	IgG/lambda	30	no	yes	yes	yes	V+T+C+D	17	VGPR
15	M/65	IIIa/II	IgG/kappa	96	yes	yes	yes	yes	V+T+C+D	5	VGPR

^*^. According to the IMWG criteria, and the evaluation was taken at the 3^rd^ month from the HDT/AuSCT;

^**^. Defined from commencement of induction treatment to HDT/AuSCT.

Abbreviations: A, doxorubicin; BM, bone marrow; C, cyclophosphamide; CA, cytogenetic abnormalities; CR, complete response; D, dexamethasone; DSS, Durie-Salmon Stage; F, female; HDT/AuSCT, high dose chemotherapy followed by autologous stem cell transplantation; ISS, International Staging System; M, male; M-protein, myeloma immunoprotein; m, month; PC, plasma cell; PR, partial response; Pt, patient; T, thalidomide; V, bortezomib; VGPR, very good partial response; yr, years; ZA, zoledronic acid.

### Characteristics of bone marrow mesenchymal stem cells and adipose tissue-derived mesenchymal stem cells

**Figure 1:**
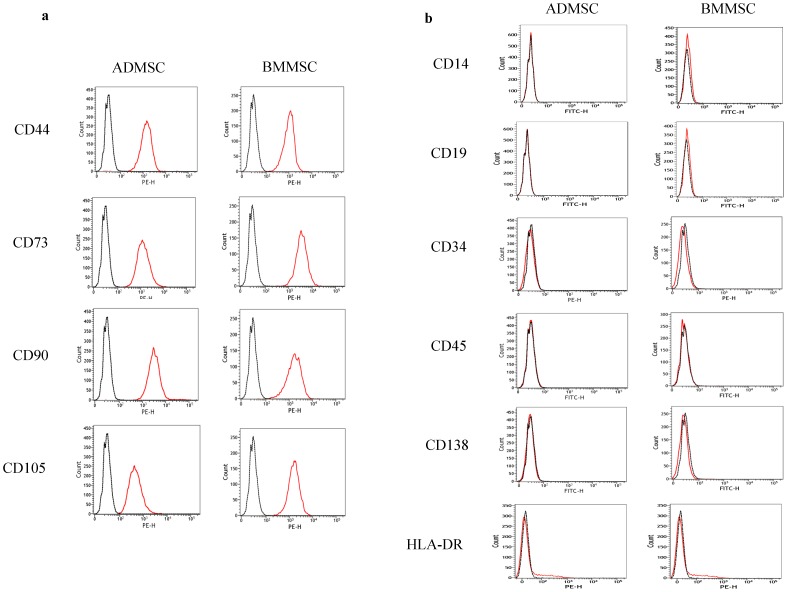
Surface marker analysis of ADMSCs and paired BMMSCs. All the ADMSCs and BMMSCs were immune-positive for the MSCs cell surface markers: CD44, CD73, CD90 and CD105 (a) but immune-negative for the hematopoietic cell surface markers: CD14, CD19, CD34, CD45, CD138 and HLA-DR (b). The results are presented as FACS histograms (isotype control stain  =  black dot line histogram; surface marker stain  =  orange solid line histogram).

All of the attached mononuclear cells harvested from subcutaneous adipose tissues and paired BM were immunopositive for CD44, CD73, CD90, and CD105 (Figure 1a), but were immunonegative for CD14, CD19, CD34, CD45, CD138 and HLA-DR (Figure 1b), which are compatible with the surface marker expression of MSCs.

### Adipose tissue-derived mesenchymal stem cells exhibited a higher expansion capacity and shorter doubling time than paired bone marrow mesenchymal stem cells did

**Figure 2:**
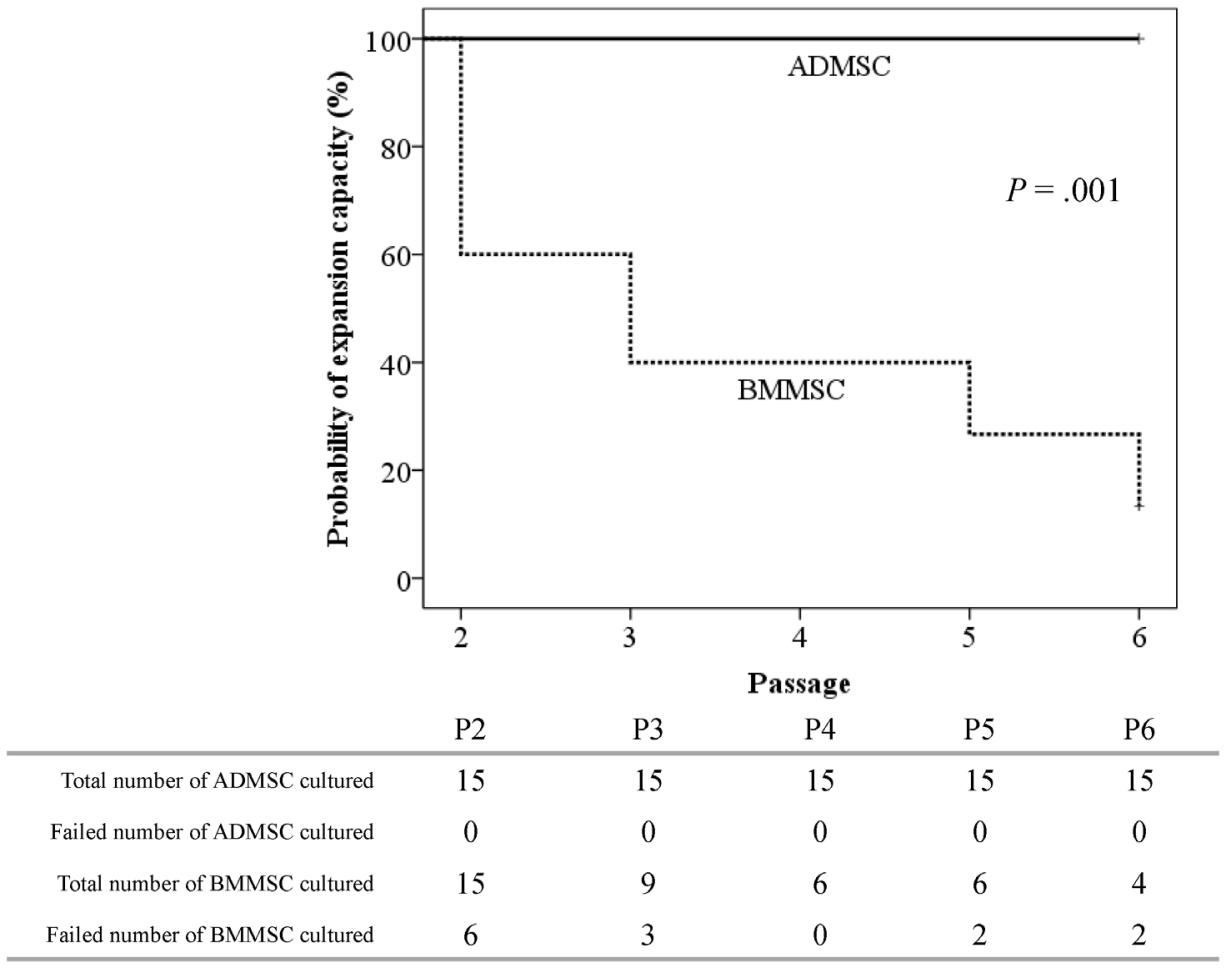
The expansion capacity of MM-ADMSCs and paired BMMSCs.

The expansion capacity of MM-ADMSCs and MM-BMMSCs from P2 to P6 were evaluated and are shown in Figure 2. The expansion capacities of cultured MM-ADMSCs were maintained at 100% from P2 to P6. By contrast, the expansion capacitiesof paired BMMSCs decreased gradually with the propagation of passages, and dropped to only 13% at P6. The difference in expansion capacities between the MM-ADMSCs and paired BMMSCs was statistically significant (*P* = .001). Notably, except for the induction duration, which was significantly longer in the failure patients (P2 failure; n = 6) than in the other patients (11.8 mo vs 6.2 mo, respectively; *P* = .001), no other clinical characteristics, including the total white blood cells (WBC), hemoglobin and platelet levels at sampling of BM and adipose tissue, as well as ALP, lactate dehydrogenase (LDH), beta-2-microglobulin (B2MG), whether response to HDC/AuSCT, presence of bone disease, and the type of induction chemotherapy, were associated with BMMSCs from failed cultures. The mean doubling times for MM-ADMSCs and paired BMMSCs were 27.7±1.6 hours and 114.9±31.5 hours, respectively, and the difference was statistically significant (*P* = .019).

### Senescence associated beta-galactosidase staining

**Figure 3:**
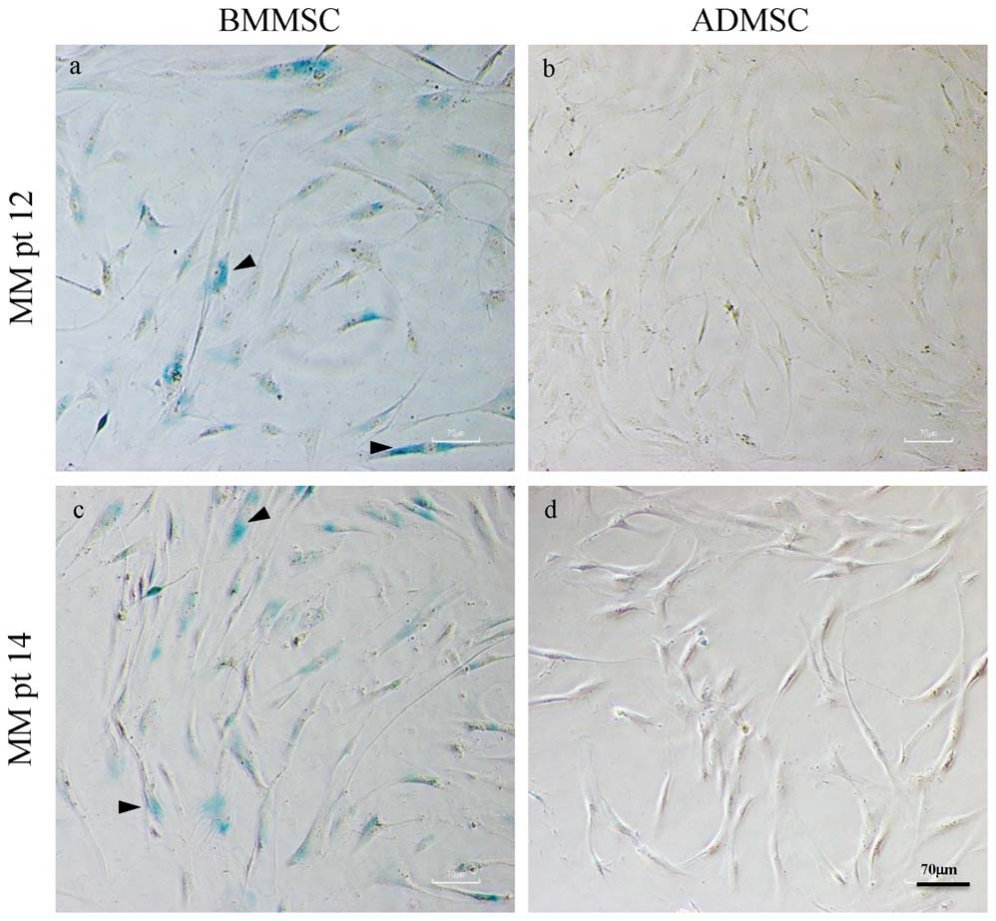
Senescence associated beta-galactosidase staining of cultured ADMSCs and BMMSCs from two representative MM patients. Positive senescence associated beta-galactosidase stainings shown by the blue granules within cytoplasms (arrowhead) were seen in most BMMSCs (a, c), but which were not seen in any of the paired ADMSCs (b, d).

Typical positive beta-galactosidase stainings were found in most of the cultured MM-BMMSCs (Figure 3a and 3c), but which were not seen in any of the paired ADMSCs (Figure 3b and 3d).

### Alkaline phosphatase activity

ALP activity of MSCs from passage 2 to 4 each measured on the Day 4 after the osteogenic induction was 0.78±0.19 U/µg for MM-ADMSCs and 0.74±0.14 U/µg for the paired BMMSCs, and the differences between the MM-ADMSCs and MM-BMMSCs were not statistically significant (*P* = .834). There was no statistically significant difference for the ALP activity on ADMSCs between healthy donors and MM patients (0.94±0.28 U/µg and 0.74±0.14 U/µg, respectively; *P* = .193), neither on BMMSCs between non-MM control and MM patients (0.77±0.23 U/µg and 0.78±0.19 U/µg, respectively; *P* = .975).

### Alizarin red S staining

**Figure 4:**
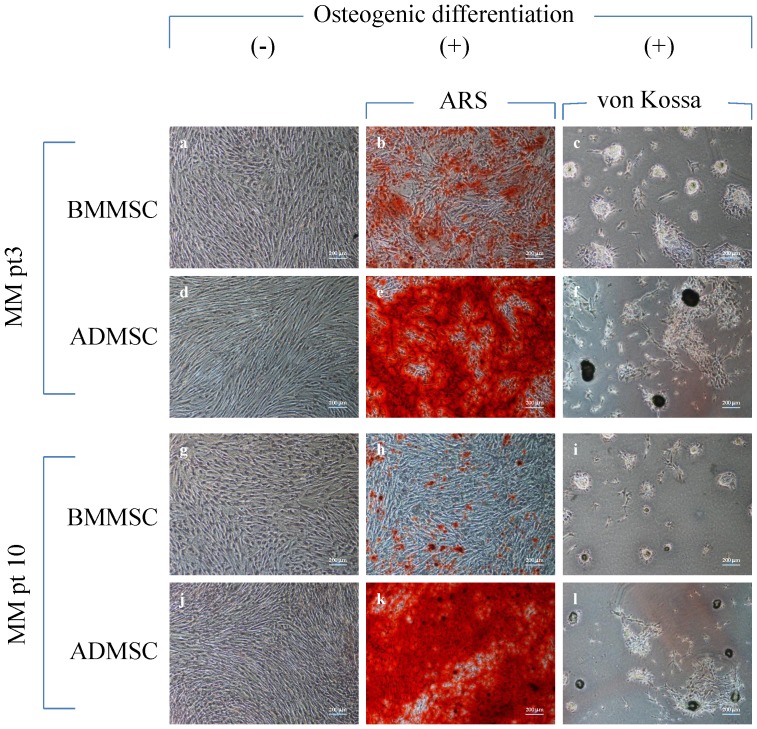
Morphologies, Alizarin Red S and von Kossa staining of cultured ADMSCs and BMMSCs from two representative MM patients. Both ADMSCs (d, j) and BMMSCs (a, g) had typical morphology for MSCs, like spindle shapes, fibroblast-like morphology and aligned in whirl formations. Positive ARS staining for calcium deposits appeared red granules for ADMSCs (e, k) and paired BMMSCs (b, h). Bone nodules were identified by the von Kossa staining and were shown in brown-black granules for ADMSCs (f, l) and paired BMMSCs (c, i).

The cultured MSCs exhibited spindle-shaped and fibroblast-like morphologies before osteogenic differentiation. No differences in the gross morphology were observed between MM-ADMSCs (Figures 4g & 4j) and paired BMMSCs (Figures 4a & 4d). Red granules appeared in positive ARS staining for calcium deposits for both the MM-ADMSCs (Figures 4e & 4k) and paired BMMSCs (Figures 4b & 4h).

### Bone nodule formation and von Kossa stain quantification

Bone nodules were identified using von Kossa staining and were shown in brown-black granules for both the MM-ADMSCs (Figures 4f & 4l) and paired BMMSCs (Figures 4c & 4i). The total von Kossa-positive area on MM-ADMSCs (2.6×10^5^ µm^2^) was greater than that on paired BMMSCs (5×10^4^ µm^2^), and the difference was statistically significant (*P* = .042).

### Quantification of mineralization

**Figure 5:**
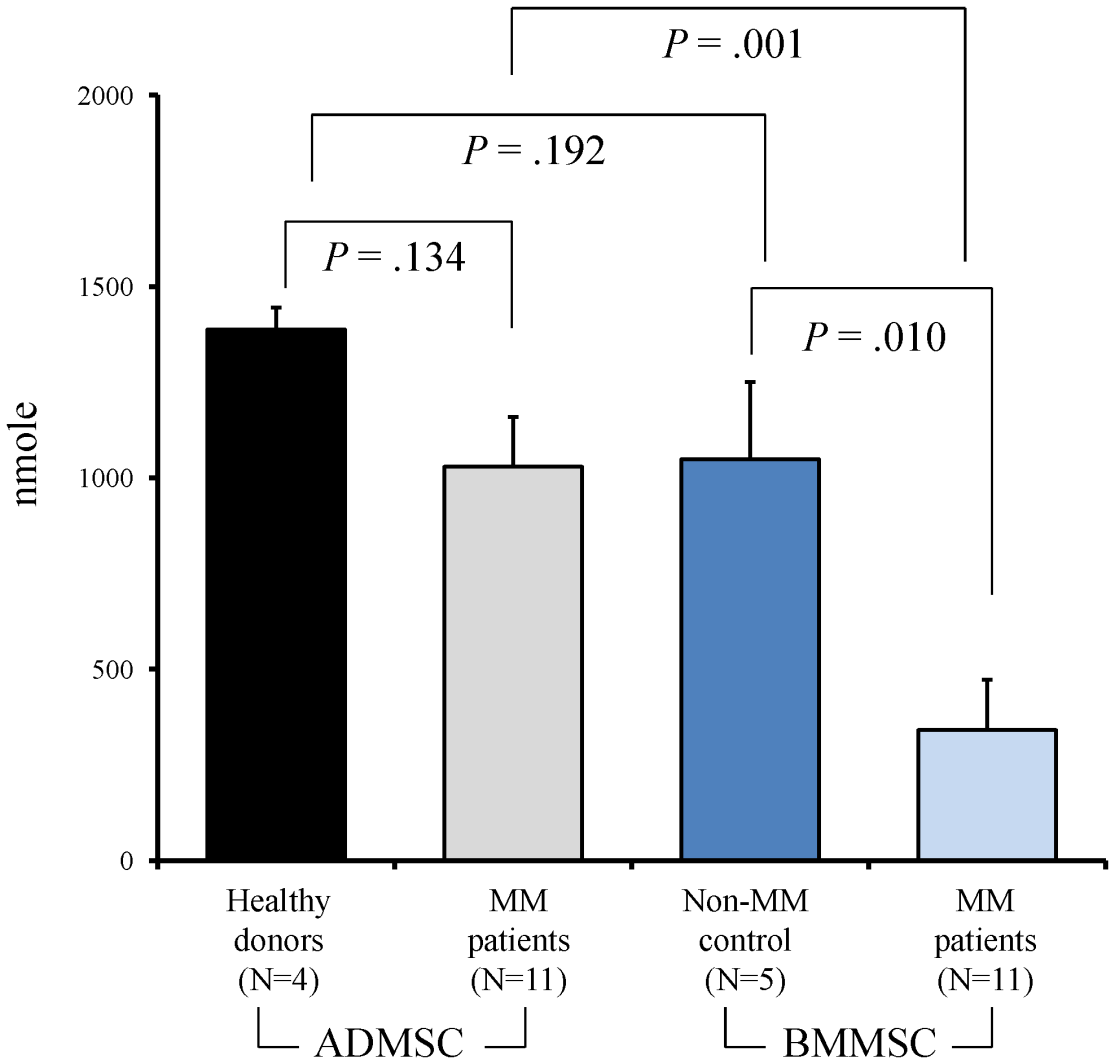
The calcium deposition levels of cultured ADMSCs and BMMSCs from healthy donors, non-MM control and MM patients.

The calcium deposition levels quantified using ARS were 1029±131 nmole for ADMSCs and 341±132 nmole for BMMSCs. The difference was statistically significant (*P* = .001). There was also significant difference on the calcium deposition between BMMSCs from myeloma patients and non-MM control (341±132 nmole vs 1049±202 nmole, respectively; *P* = .010), but on contrast, no significant difference between ADMSCs from healthy donors and MM patients (1388±57 nmole and 1029±131 nmole, respectively; *P* = .134) (Figure 5).

## Discussion

Based on our thorough review of relevant literature, this is the first study to evaluate osteoblastogenesis from various MSCs sources, namely paired ADMSCs and BMMSCs, in MM patients who had completed novel-agent-based induction and standard intensive HDT. Our study supports the hypothesis that ADMSCs are a more favorable MSC source for osteoblastogenesis than BMMSCs, because of their higher expansion capacity, shorter doubling time in cell cultures, greater calcium deposition ability and greater osteoblastic activity. In line with our findings, several studies have provided evidence that ADMSCs exhibit advantages over BMMSCs in neural differentiation and myogenesis [32], [33].

The sampling of the MSCs was chosen at post- intensive therapy based on the assumption that the impaired osteoblastogenesis in MM patients resulted from the MCs as well as anti-MM therapy [18], [34], [35]. Therefore, we determined whether BM-targeted therapy in MM affects ADMSCs. Compared to the ADMSCs obtained from healthy donors (n = 4), the osteoblastogenesis potential of the posttherapy MM-ADMSCs did not differ significantly suggesting that MM-ADMSCs might not be affected by MCs and anti-MM treatment. By contrast, the osteogenic differentiation, esp. the calcium deposition, of post-therapy MM-BMMSCs was significantly impaired compared with non-MM control (n = 5; Figure 5). Furthermore, similar to others [35], sign of senescence (ex. positive beta-galactosidase staining) was seen in our MM-BMMSCs, but which was not seen in paired ADMSCs (Figure 3). The underlying mechanism for these observed differences between MM-ADMSCs and MM-BMMSCs is not yet clear; the difference might be due to intrinsic differences between MM-ADMSCs and MM-BMMSCs caused by patients' ages, MCs, and the BM-targeted therapy [35]. To partly support this explanation, the MM-BMMSC cultures that failed at P2 were associated with a longer duration of clinical induction treatment than MM-BMMSCs cultures that propagated beyond P2. Unfortunately, our data was not sufficient to realize how much impact from the anti-MM treatment on the dysfunction of BMMSC, since there was lack of sequential follow-up of the clinical samples at different time points, namely, at diagnosis, after induction, and post HDC/AuSCT; therefore, further studies are required. The BMMSCs and the ADMSCs can be cryopreserved before commencing anti-MM treatment; however, cryopreservation requires a large storage facility and huge expense to accommodate BMMSCs or ADMSCs for long periods [36]. In addition, certain genetic aberrations have been observed in MM-BMMSCs that are likely to impair the osteogenic differentiation pathways, such as the Wnt and Notch signaling pathways [37], [38]. These changes have been reported to persist in BMMSCs from MM patients after extended culture and passage in vitro, indicating that these MM-BMMSCs might be permanently modified [17], [18], [34], [39]. These genetic aberrations might limit the use of BMMSCs in MM patients. Therefore, confirming that ADMSCs can differentiate into functional OBs in MM patients who have received intensive treatment and exhibited the optimal response is clinically relevant. Because MSC treatment for various degenerative diseases requires a large quantity of autologous MSCs, good-manufacturing-practices (GMP)-compliant large-scale ex vivo expansion is essential for future therapeutic purposes [40]; therefore, a favorable MSC source exhibits high viability and potency in an in vitro expansion. The number of BMMSCs in the BM is approximately 1 in 25,000 to 100,000 nucleated cells; however, the average number of ADMSCs in processed lipoaspirate is approximately 2 in 100 nucleated cells [41]. Overall, ADMSCs might be a more suitable MSC source than BMMSCs for clinical use in the future.

Our data also showed that the early osteogenic differentiation capacity determined according to the ALP activity did not differ significantly between the 2 paired MSCs, and this finding was also seen between the BMMSCs from MM patients and the non-MM control, suggesting that MCs and/or the related anti-MM therapy primarily affect the late mineralization stage of the osteogenic differentiation of MSCs. Further detailed study, such as molecular profiling of the various stages of differentiation from MSCs to OBs, is required to validate this observation.

This study had several limitations. First, the cohort was small; therefore, the observations must be validated in studies enrolling more patients. However, the paired ADMSCs and BMMSCs obtained from a nearly homogenous patient population who had received uniform treatment procedures provided a great opportunity to minimize the bias caused by the various clinical features of patients, and our data consistently showed that the ADMSCs are a more favorable MSC source than BMMSCs for future cell therapy for MMBD. Second, this study enrolled only relatively young MM patients who could tolerate the HDT/AuSCT. Therefore, we must confirm whether the ADMSCs from elderly MM patients, who are likely to have more prevalent osteopenia and MMBD, are a favorable source for MSC cell therapy. A recent report has indicated that ADMSCs are less affected by patient age than BMMSCs are [24]. Third, the greater calcium deposition ability and osteoblastic activity of ADMSCs compared with BMMSCs did not necessarily indicate that ADMSCs have greater ability to repair MMBD. In the 2-dimensional culture, we observed that, after osteogenic induction, the MSCs differentiated into OBs and formed osteoid and calcification, but whether these calcified osteoids can heal bone destruction or improve bone strength is unclear. The recent development of 3-dimensional cultures using biological scaffold material, which allows the growth of MSCs within a stereotypical structure [42], will provide the opportunity to assess the ability of building real bony structures by using these 2 MSCs in MM patients.

In conclusion, our findings confirm that osteoblastogenesis can be induced in ADMSCs of MM patients who have received intensive treatment, and that the ADMSCs are a feasible and more favorable MSC source than BMMSCs for future cell therapy for MMBD.
